# Deep learning-based detection of focal cortical dysplasia in children: External validation of the MELD Graph and 3D-nnUNet pipelines

**DOI:** 10.1016/j.ynirp.2026.100410

**Published:** 2026-09-16

**Authors:** Andrea Dell’Orco, Enrico De Vita, Felice D'Arco, Annalena Lange, Theodor Rüber, Angela M. Kaindl, Mike P. Wattjes, Ulrich-Wilhelm Thomale, Lena-Luise Becker, Anna Tietze

**Affiliations:** a Charité-Universitätsmedizin Berlin, Corporate Member of Freie Universität Berlin and Humboldt-Universität Berlin, Institute of Neuroradiology, Berlin, Germany; b MR Physics Group. Radiology, Great Ormond Street Hospital for Children NHS Foundation Trust, London, United Kingdom; c Developmental Imaging and Biophysics Section, Developmental Neurosciences, UCL Great Ormond Street Institute of Child Health, University College London, London, United Kingdom; d Department of Neuroradiology, University Hospital Bonn, Bonn, Germany; e Department of Epileptology, University Hospital Bonn, Bonn, Germany; f Institute for Computer Science, University of Bonn, Bonn, Germany; g German Center for Neurodegenerative Diseases (DZNE), Bonn, Germany; h Center for Medical Data Usability and Translation (ZMDT), University of Bonn, Bonn, Germany; i Center for Chronically Sick Children, Institute of Cell Biology and Neurobiology, Charité-Universitätsmedizin Berlin, Corporate Member of Freie Universität Berlin and Humboldt-Universität Berlin, Dept. of Pediatric Neurology, Berlin, Germany; j Charité-Universitätsmedizin Berlin, Corporate Member of Freie Universität Berlin and Humboldt-Universität Berlin, Dept. of Pediatric Neurosurgery, Berlin, Germany

**Keywords:** Focal cortical dysplasia, nnUNet, MELD Graph, Pediatric epilepsy, Lesion detection, External validation

## Abstract

**Objective:**

To externally validate two recently developed deep-learning approaches for FCD detection, MELD Graph and 3D-nnUNet, in a pediatric cohort.

**Background:**

Focal cortical dysplasias (FCDs) are among the most common structural causes of drug-resistant epilepsy in children but are frequently subtle and difficult to detect on MRI. Several automated lesion detection methods have been proposed to support neuroradiological assessment. We externally validated two recently developed deep-learning approaches for FCD detection, MELD Graph and 3D-nnUNet, in a pediatric cohort.

**Methods:**

In this retrospective single-center study, we analyzed brain MRI scans of 70 children evaluated for epilepsy, including 34 MRI-positive patients with suspected FCD and 36 MRI-negative patients, based on primary radiology reports. Both models were applied to 3D T1-weighted and 3D FLAIR images. The detected lesions were reviewed by an experienced pediatric neuroradiologist and classified as true positive, false positive, or false negative. False-positive cases were additionally reviewed using electroclinical findings.

**Results:**

MELD Graph and 3D-nnUNet achieved comparable lesion precision (0.86 vs. 0.90) but only moderate recall (0.63 vs. 0.47). At the patient level, MELD Graph demonstrated higher sensitivity (0.71 vs. 0.53), whereas 3D-nnUNet achieved substantially higher specificity (0.86 vs. 0.47) owing to fewer false-positive detections. 3D-nnUNet showed higher sensitivity with the improved FLAIR sequence, whereas MELD Graph performance was strongly influenced by the accuracy of cortical surface reconstruction. Both models failed to detect a clinically relevant proportion of FCDs.

**Conclusion:**

MELD Graph and 3D-nnUNet generalize to pediatric MRI and represent valuable decision-support tools for highlighting suspicious cortical regions. However, their moderate sensitivity and susceptibility to false-positive findings indicate that they should complement, rather than replace, expert neuroradiological interpretation.

## 1 Introduction

Epilepsy remains a major cause of morbidity and mortality in childhood (Aaberg et al., 2017; Biset et al., 2024). Approximately 25% of cases have structural causes, including perinatal or acquired brain injury, low-grade tumors, or vascular malformations, whereas approximately 5% are related to malformations of cortical development (Aaberg et al., 2017). Among these, focal cortical dysplasias (FCDs) are the most common (Blümcke, 2024). FCDs result from disordered neuronal maturation and cortical organization, are highly epileptogenic, and are histologically classified into three groups, with FCD type II being the most common subtype (Blümcke, 2024). In a large study by Blümcke et al., specimens from more than 2600 children undergoing epilepsy surgery were histopathologically analyzed, and approximately 28% were found to have FCDs (Blümcke, 2024). However, FCDs are often subtle and can be difficult to detect on conventional MRI. This underscores the importance of evaluating lesion detection methods specifically in the pediatric population; however, such studies remain scarce (Hom et al., 2024).

Antiseizure medication (ASM) is the first-line treatment, but approximately one-third of children develop drug-resistant epilepsy, meaning that they continue to have seizures despite adequate trials of at least two ASMs (Sultana et al., 2021). For children with drug-resistant epilepsy, epilepsy surgery can be an effective treatment option, with the potential to achieve complete seizure freedom or reduce seizure frequency, which may allow ASM dose reduction or even discontinuation. Successful surgical treatment can profoundly improve neurodevelopmental and cognitive outcomes as well as the quality of life of both the child and the family (Cross et al., 2022). Identifying epileptogenic lesions is therefore crucial for determining whether surgery is a viable option.

Multimodal approaches have been proposed to improve diagnostic accuracy, including arterial spin labeling (Pastore et al., 2024), edge-enhanced MP2RAGE (Magnetization Prepared Rapid Acquisition Gradient Echo with two inversion times) (Middlebrooks et al., 2020), positron emission tomography (PET)-based techniques, and single-photon emission computed tomography (SPECT) (von Oertzen et al., 2023). In addition, advanced post-processing of MRI data has become an important and evolving strategy to detect subtle abnormalities and reduce the number of MRI-negative cases. Different approaches have been described, including voxel-based morphometry (Huppertz et al., 2005), voxel-based intensity analysis (Focke et al., 2008), and sulcal morphometry (Bernasconi et al., 2011; Rivière et al., 2002).

Automated FCD detection is attractive because it is relatively easy to implement in clinical practice and typically relies on standard MRI sequences such as 3D T1-weighted and FLAIR images. Since manual detection can be challenging and time-consuming, these tools offer valuable aid in the daily radiological workflow. However, the sensitivity and specificity must be carefully balanced: missing a lesion is unacceptable, yet low specificity with excessive false positives increases the number of findings that require manual verification, ultimately reducing radiologist efficiency. Postprocessing methods have been validated against human readers, with mixed results (David et al., 2021; Kersting et al., 2025; Son et al., 2023).

To address this challenge, the multicenter lesion detection (MELD) consortium has recently refined its machine learning–based approach by incorporating graph neural networks (MELD Graph, Ripart et al., 2025), which has significantly improved positive predictive values (PPVs) in a large cohort of more than 500 patients. Moreover, a recent study (Kersting et al., 2025) suggested voxelwise convolutional neural network–based segmentation using nnU-Net architectures (hereafter referred to as 3D-nnUNet). Both methods are promising because they lead to a high detection rate while maintaining a low number of false positive results.

Although both methods are evaluated on a multicenter cohort, the datasets consisted predominantly of adult patients. Each method has previously been evaluated on small external pediatric cohorts of 9–10 patients each (De Vita et al., 2025; Kersting et al., 2025). Therefore, comprehensive external validation in a larger pediatric population remains essential to reliably assess their performance.

The aim of our study was to validate MELD Graph and 3D-nnUNet approaches in a pediatric population by comparing children with FCD to those with MRI-negative examinations performed because of seizures and/or abnormal EEG findings. The study was designed as a clinically oriented lesion-detection and patient-classification study, with the specific aim of evaluating whether these methods can serve as visual guidance tools by highlighting suspicious cortical regions that warrant closer inspection, while leaving the final diagnostic interpretation to the radiologist. We hypothesized that both methods would perform comparably to the original publications, with a number of false-positive findings acceptable for the radiological workflow, and that their performance would depend on MRI data quality.

## 2 Methods

### 2.1 Dataset

The study was approved by the local Ethics Committee at Charité (EA2/295/25) and was conducted according to the guidelines of good clinical practice. Informed consent was waived in this retrospective study by the local Ethics Committee.

We searched our clinical picture archiving and communication system (PACS) retrospectively from July 2019 to March 2025 to identify MRI studies of patients evaluated for seizures or epilepsy. We included all patients in whom the original clinical radiology report described a FCD (MRIpos cases). These reports were issued by one of the three board-certified neuroradiologists responsible for pediatric imaging at our institution, all of whom have extensive experience in pediatric epilepsy MRI. We then selected an equal number of MRI-negative cases (MRIneg), defined as examinations in which no structural epileptogenic lesion was identified in the original clinical report and where the same imaging protocol had been employed (from March 2025 backwards). MRIneg patients had undergone MRI because of seizures, abnormal EEG findings, or suspected brain malformations. MRI images were acquired on a Skyra 3T scanner (Siemens Healthineers, Erlangen, Germany) with a dedicated 64-channel head coil and an MRI protocol including sagittal 3D T1w (MPRAGE) and 3D FLAIR. The MRI acquisition parameters are reported in Table 1.

**Table 1:** MRI acquisition parameters.

	MPRAGE	FLAIR278	FLAIR250
**field of view (mm)**	240	230	230
**TR/TE/TI (ms)**	2300/2.32/900	4500/387/1800	6000/449/1800
**flip angle (∘)**	8	variable	variable
**base resolution**	256	256	256
**turbo factor**	224	278	250
**acquisition time**	5 min 21 s	5 min 12 s	7 min 44 s
**Bandwidth (Hz/Px)**	200	751	751
**Voxel Size (mm^3^)**	0.9 × 0.9 × 0.9	0.9 × 0.9 × 0.9	0.9 × 0.45 × 0.45

From 2024 onward, an improved 3D FLAIR sequence was used, in which the turbo factor was decreased from 278 to 250 (hereafter referred to as FLAIR278 and FLAIR250, respectively), and the voxel size was reduced. The MRI parameters for the 3D T1w images and the two versions of the 3D FLAIR sequences are provided in Table 1. In addition, defaced example NIfTI files for the two sequences used in this study are available for download (Dell’Orco and Tietze, 2026). Only data free of motion artifacts or artifacts due to dental braces were included.

In addition to the MRI data, the original radiology report was retrieved. Patients with structural epileptogenic lesions other than FCD, including isolated polymicrogyria, pachygyria, and epileptogenic tumors that were clearly recognized as such on the initial MRI, were excluded from the analysis. Importantly, patients who were part of the original MELD training set were excluded.

### 2.2 MELD Graph segmentation

DICOM images exported from PACS were converted into NIfTI format with dcm2niix (Li et al., 2016) and sorted in BIDS (Gorgolewski et al., 2016) format. A different harmonization model was used for each FLAIR version. For all patients, the harmonization step of the MELD Graph pipeline (v2.2.5) was run using the --harmo_only flag, which also performs the required FreeSurfer (Fischl, 2012) recon-all segmentation, followed by the lesion prediction step.

Because incorrect FLAIR-to-T1w coregistration or poor FreeSurfer segmentation may affect lesion detection, the quality of coregistration, segmentation, and pial surface reconstruction was visually assessed for misregistration and missegmentation.

Quality control combined automated metrics obtained with the FSQC (Esteban et al., 2017) tool, a quality-assurance and quality-control toolkit for FreeSurfer- and FastSurfer-processed structural MRI data that provides quantitative image- and segmentation-quality metrics, with subsequent visual inspection. FSQC, which was previously used in a similar study (Kaas et al., 2026), has shown high agreement with expert ratings in a validation study (Wong, 2026). Visual quality control was then performed independently by A.T. and A.D. following the MELD QC protocol (Adler-Wagstyl et al., 2018). Each subject was visually inspected and assigned a score of 1 (good segmentation), 2 (borderline), or 3 (to be excluded). Disagreements were resolved by consensus. Particular attention during visual review was given to subjects showing multiple outlying FSQC metrics based on z-scores of the individual metrics. Example images from the quality-control procedure are provided in the Supporting Information, Figs. S2 and S3.

As an additional sensitivity analysis, the MELD Graph pipeline was also run using T1w images only, without FLAIR input, and evaluated using the same lesion- and patient-level performance metrics as the main T1w + FLAIR analysis.

### 2.3 3D-nnUNet

To obtain the final predictions, we adopted the same ensemble strategy as described in the original publication (Kersting et al., 2025). FLAIR images were rigidly coregistered to the T1w images using ANTs (Avants et al., 2008), using the default linear interpolation for resampling. The coregistered T1w and FLAIR images were then arranged into a nnU-Net-compatible dataset and segmented using the five independently trained models. The five model outputs were averaged voxel-wise, and the final segmentation was obtained by binarizing the resulting map at a threshold of 0.5, corresponding to a majority-vote rule across the ensemble. For this analysis, we used a conda environment with nnU-Net 2.6.0 and the publicly released model from GitLab commit 992f8cd5 (https://gitlab.com/lab_tni/projects/nnunet_fcd). Both pipelines were applied once per subject using the publicly released pretrained models. No retraining or repeated inference runs with different random seeds were performed. For 3D-nnUNet, the five pretrained models were combined using the ensemble strategy described above.

### 2.4 Neuroradiological and clinical evaluation of the detected lesions

The MELD Graph pipeline generates a PDF report highlighting each detected lesion together with a corresponding segmentation mask in NIfTI format. Similarly, 3D-nnUNet produces lesion segmentation masks in the NIfTI format. Lesions were annotated by anatomical location and hemisphere. MRI scans were initially assessed in routine clinical practice by the pediatric neuroradiologist on duty. All patients were subsequently re-evaluated by an experienced pediatric neuroradiologist (A.T.) with more than 10 years of experience in pediatric epilepsy imaging. This review compared the initial clinical reports with the MELD Graph and 3D-nnUNet segmentation masks, which are displayed as overlays on T1w and FLAIR images as well as all available MRI sequences in the PACS.

The lesions were classified as follows:

1. True positive lesions (TPL): identified by the neuroradiologist and detected by the model.
2. False positive lesions (FPL): detected by the model but not identified by the neuroradiologist as lesions, even after thorough re-evaluation.
3. False negative lesions (FNL): identified by the neuroradiologist but not detected by the model.
4. Retrospectively identified candidate lesions (rCL), initially overlooked by the neuroradiologist but recognized on re-evaluation after review of the lesion-detection algorithm output. These retrospectively identified lesions were therefore reported separately and not incorporated into the predefined reference standard used for formal performance calculations.

Patient records with FPLs were re-evaluated by an experienced pediatrician specializing in pediatric neurology with extensive experience in pediatric epileptology (L.-L.B.). Clinical semiology and EEG findings were reviewed and compared with the results, with particular attention given to whether the identified lesion was consistent with the clinical presentation.

### 2.5 Statistical evaluation

Statistical evaluation was performed separately for the two methods using the same set of metrics. Two complementary tasks were defined: a lesion-detection task and a patient-classification task.

The lesion-detection task is the primary endpoint and evaluated the ability of the methods to localize individual lesions defined by neuroradiological assessment while minimizing FPLs that are time-consuming to review. In MRIpos patients, performance was quantified using lesion-level precision (also called positive predictive value, PPV) and recall (also called sensitivity). In MRIneg patients, no true lesions exist by definition; therefore, evaluation of the lesion-detection task was limited to quantifying FPL detections, summarized as the number of FPL per patient. Including MRIneg cases in lesion-level precision would be misleading, as they can contribute false positives but not true positives.

The patient-classification task was formulated as a binary decision problem, assessing whether a scan contained at least one lesion that was correctly detected by the model. Sensitivity and specificity were calculated based on the neuroradiological ground truth described above. At the patient level, true-positive patients (TPP) were defined as MRI-positive patients with at least one true lesion detected by the model; false-negative patients (FNP) were defined as MRI-positive patients without any detected true lesion; false-positive patients (FPP) were defined as MRI-negative patients with at least one incorrect detection; and true-negative patients (TNP) were defined as MRI-negative patients without false-positive detections. Although recall and sensitivity are mathematically equivalent, we use the terms recall for lesion-level detection and sensitivity for patient-level classification throughout the manuscript to avoid confusion between the two tasks. For each performance metric, 95% confidence intervals (CIs) were estimated using non-parametric bootstrapping with 1000 resamples.

Additionally, to investigate differences between the models and FLAIR sequences, we fitted four regression models. For the lesion-detection task, we used a mixed-effects logistic regression with correctly detected lesions as the outcome (TPL = 1, FNL = 0), the model as a predictor, and the FLAIR sequence, patient age, and sex as covariates. The subject was included as a random effect. For the patient-classification task, we used a logistic regression with correctly classified patients as the outcome and the same predictors and covariates.

To further assess the effect of the two different FLAIR sequences on patient-level errors, we fitted two additional logistic regression models with false-positive patient status and false-negative patient status as outcomes. Both models included the model, FLAIR sequence, and their interaction as predictors. A p-value smaller than 0.05 was considered statistically significant. No correction for multiple testing was applied, as the regression analyses were considered exploratory.

Formal definitions and formulas for all performance metrics are provided in Supporting Information S1.

## 3 Results

### 3.1 Dataset

**Fig. 1:**
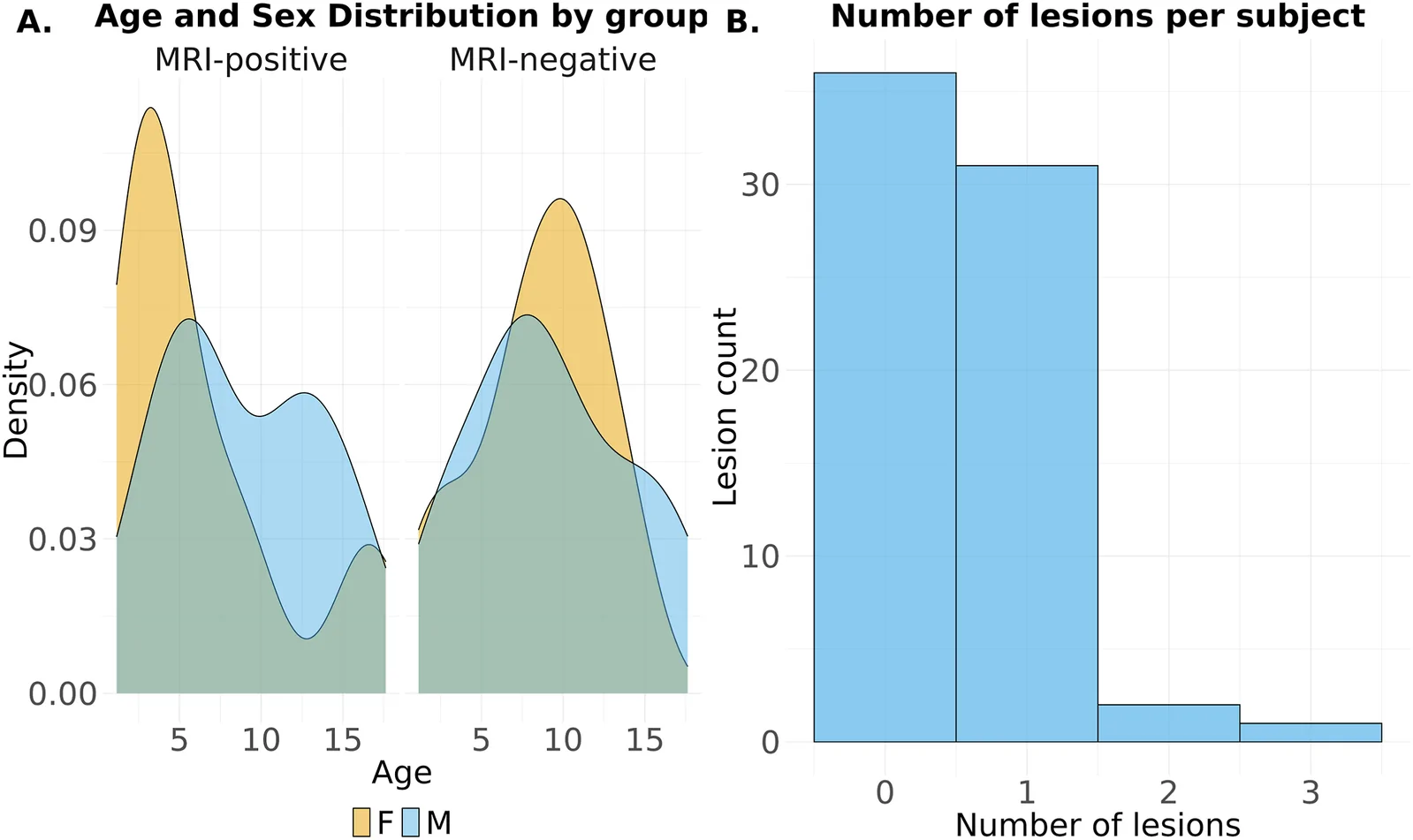
(A) Age and sex distributions (F: female, M: male) of MRI-positive (MRIpos) and MRI-negative (MRIneg) patients. B. Distribution of the number of lesions per subject across the MRIpos cohort, illustrating that most patients present with a single lesion, whereas a small subset presents with multiple lesions.

The final dataset comprised 36 MRIneg and 34 MRIpos patients, with a total of 38 ground truth lesions. One initially included MRIpos patient was excluded following detailed image review because the predominant imaging abnormality represented a gyral malformation rather than a FCD. A second patient was excluded because of a diagnosis of tuberous sclerosis complex. This exclusion was implemented in accordance with the recommendations of the latest MELD Graph version, which became available after completion of the original analysis. Among the MRIpos patients, 8 were histologically confirmed (3 FCD type 2A, 4 FCD type 2B, one FCD type 3A). In addition, two lesions were gangliogliomas, one lesion represented gliotic tissue, and one case remained histologically inconclusive. These patients were intentionally retained in the cohort because they reflect the real-world diagnostic challenge of distinguishing FCD from morphologically similar epileptogenic lesions on MRI. The remaining lesions were diagnosed on the basis of imaging alone. The cohort demographics are summarized in Table 2 and Fig. 1. Among all the patients, 43 patients (24 MRIpos, 19 MRIneg) were imaged with FLAIR278, and 27 were imaged with FLAIR250 (10 MRIpos, 17 MRIneg). A two-sided *t*-test revealed no significant difference in age between participants who were scanned with FLAIR278 and those who were scanned with FLAIR250 (t = −0.91, p = 0.36).

**Table 2:** Demographics.

	MRI-positive	MRI-negative	total
**N**	34	36	70
**Age (years, median [IQR])**	5.42 [3.22-10.93]	8.77 [6.48-11.69]	7.48 [3.63-11.62]
**% female**	58.82	50.00	54.28

### 3.2 Quality control of FreeSurfer segmentations

Following QC, five patient scans were rated as borderline (score 2), including three MRIpos (aged 1, 2 and 2 years) and two MRIneg cases (aged 1 and 7 years). These cases accounted for three FPLs, two FNLs, and one TPL. No exclusions were made on the basis of the QC. FSQC metrics and QC examples are reported in Supporting Figs. S1–3.

Additionally, to evaluate the potential impact of excluding these borderline cases, we additionally report lesion-based and patient-based analyses with these subjects excluded in Supporting Information Fig. S4.

### 3.3 Neuroradiological and clinical re-evaluation following MELD Graph analysis

**Fig. 2:**
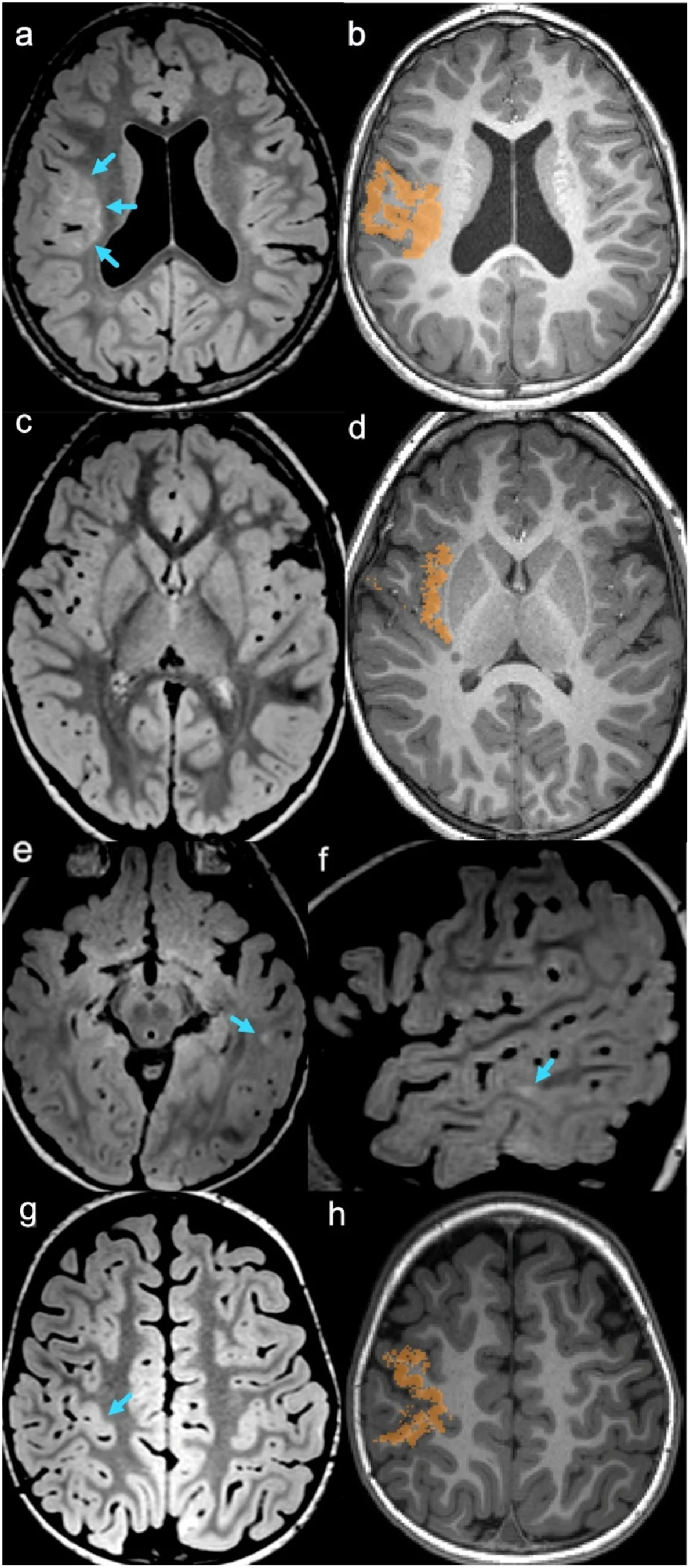
Representative imaging examples (MELD Graph): (a–b) Correctly identified lesion (TPL) in the right insula consistent with presumed FCD type 2A, shown on FLAIR (blue arrows, a) and the corresponding MELD Graph lesion mask on 3D T1-weighted MRI (b). (c–d) False-positive MELD Graph detection (FPL) (d) with the corresponding FLAIR image (c). (e–f) Lesion missed by MELD Graph (FNL), visible on FLAIR (blue arrows). (g–h) Retrospectively identified candidate lesion on FLAIR (rCL) (blue arrow, g) with the corresponding MELD Graph mask on 3D T1-weighted MRI (h).

Agreement between MELD Graph and the primary report was observed for 24 lesions (TPLs), whereas MELD Graph identified 25 lesions that could not be verified by the neuroradiologist (FPLs). Furthermore, 14 lesions were detected by the neuroradiologist but not by MELD Graph (FNLs). Importantly, two rCL were identified, lesions initially overlooked by the neuroradiologist but were considered plausible upon re-evaluation. rCL on post hoc re-evaluation were reported descriptively but were not incorporated into the predefined reference standard and therefore were excluded from formal performance calculations. Examples for each category are given in Fig. 2.

The EEG findings and clinical semiology of patients with FPL and rCL results were re-evaluated. In a patient with a left frontal FPL detected by MELD Graph, the EEG demonstrated a central focus that was considered potentially concordant with the MELD Graph finding. In the remaining FPL cases, EEG and semiology were not consistent with the MELD Graph-identified lesions. Similarly, both rCL cases showed no concordance between the imaging findings and the clinical data.

**Fig. 3:**
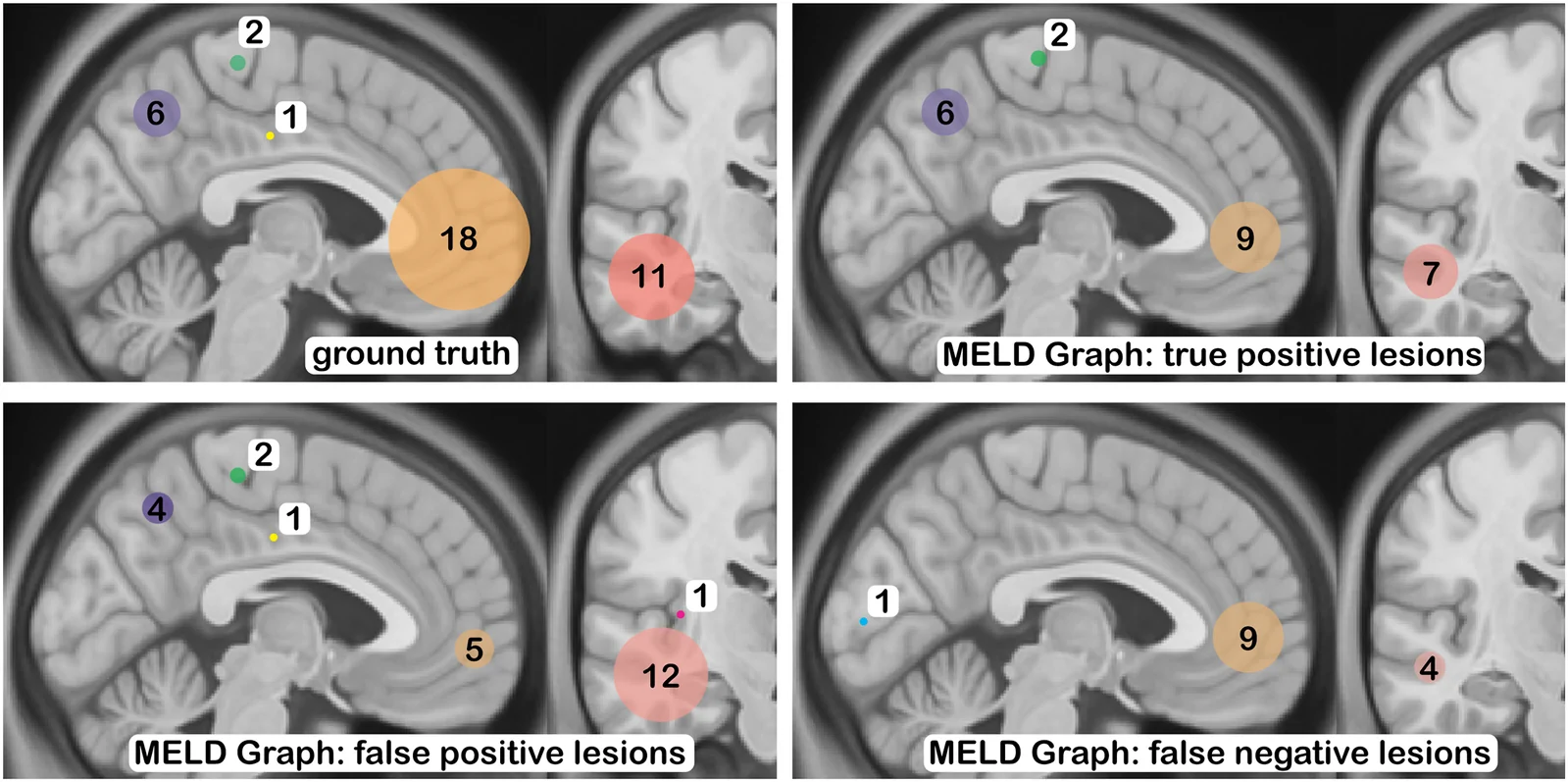
Distribution of ground-truth lesions on the basis of the original radiology reports and the corresponding true positive, false positive, and false negative detections generated by MELD Graph, stratified by anatomical location (orange: frontal; light red: temporal; purple: parietal; green: frontoparietal; turquoise: occipital; yellow: cingulate, red: insula).

Among the TPLs, most were located in the frontal lobe (n = 9), followed by the temporal (n = 7), parietal (n = 6), and frontoparietal regions (n = 2). Considering both the MRIpos and MRIneg patients together, the 25 FPL were found in the temporal (n = 12), frontal (n = 5), frontoparietal (n = 2) and parietal (n = 4) regions, with single occurrences in the insula and cingulate. FNL occurred predominantly in the frontal lobes (n = 9), followed by the temporal lobe (n = 4), with one case in the occipital lobe. The two rCL were in the frontoparietal and frontal lobes. The overall distribution is illustrated in Fig. 3.

The MELD Graph T1w-only sensitivity analysis showed lower lesion-level precision and recall, whereas the false-positive lesion burden in MRI-negative patients was similar to that of the main T1w + FLAIR analysis. Detailed results are reported in Supporting Information Fig. S5 and Table 3.

**Table 3:** Comparison of performance metrics between the present study and previous similar studies. Notably, part of the variability across studies may also reflect differences in cohort definitions. CI, confidence interval. (1) Recalculated from aggregate counts kindly provided by the original authors to align the metrics with those used in the present study. In the original paper, MRI-positive and MRI-negative cases were analyzed together.

Study	This study			Ripart et al. (2025)	Kersting et al. (2025)	De Vita et al. (2025)	Kaas et al. (2026)
**Model**	MELD Graph T1w + FLAIR	MELD Graph (T1w-only)	3D-nnUNet	MELD Graph	3D-nnUNet	MELD Graph	MELD Graph
Lesion Detection Task - MRIpos							
**Precision/PPV**	0.86	0.75	0.90	0.76	0.63	0.58	0.63 (1)
**95% CI**	0.72-0.96	0.60-0.91	0.75-1.00	0.61-0.79	-	-	-
**Recall/Sensitivity**	0.63	0.55	0.47	-	0.55	0.65	0.31(1)
**95% CI**	0.47-0.78	0.41-0.70	0.31-0.62	-	-	-	-
Lesion Detection Task – MRIneg							
**FPL/patient**	0.58	0.58	0.14	-	-	-	0.66
95% CI	0.39-0.77	0.39-0.81	0.02-0.25	-	-	-	-
Patient Classification Task							
**Sensitivity**	0.71	0.62	0.53	0.72	0.64	-	0.76
**95% CI**	0.55-0.85	0.45-0.77	0.36-0.69	0.61-0.78	-	-	0.67-0.84
**Specificity**	0.47	0.47	0.86	0.56	0.86	-	0.47
**95% CI**	0.31-0.64	0.30-0.63	0.75-0.96	0.47-0.66	-	-	0.38-0.56

### 3.4 Neuroradiological and clinical re-evaluation following 3D-nnUNet analysis

The model correctly identified 18 of the 38 ground-truth lesions. These TPLs were in the frontal (n = 9), temporal (n = 5), parietal (n = 3), and frontoparietal lobes (n = 1). In addition, the model produced 7 FPLs, including four in the frontal lobe and one in the insular region, as well as two detections erroneously located outside the brain. A total of 20 of the 38 lesions were missed by the model and classified as FNL. These were in the frontal (n = 8), parietal (n = 4), and temporal lobes (n = 6), with single lesions in the frontoparietal region and the cingulate cortex. No rCL were identified.

**Fig. 4:**
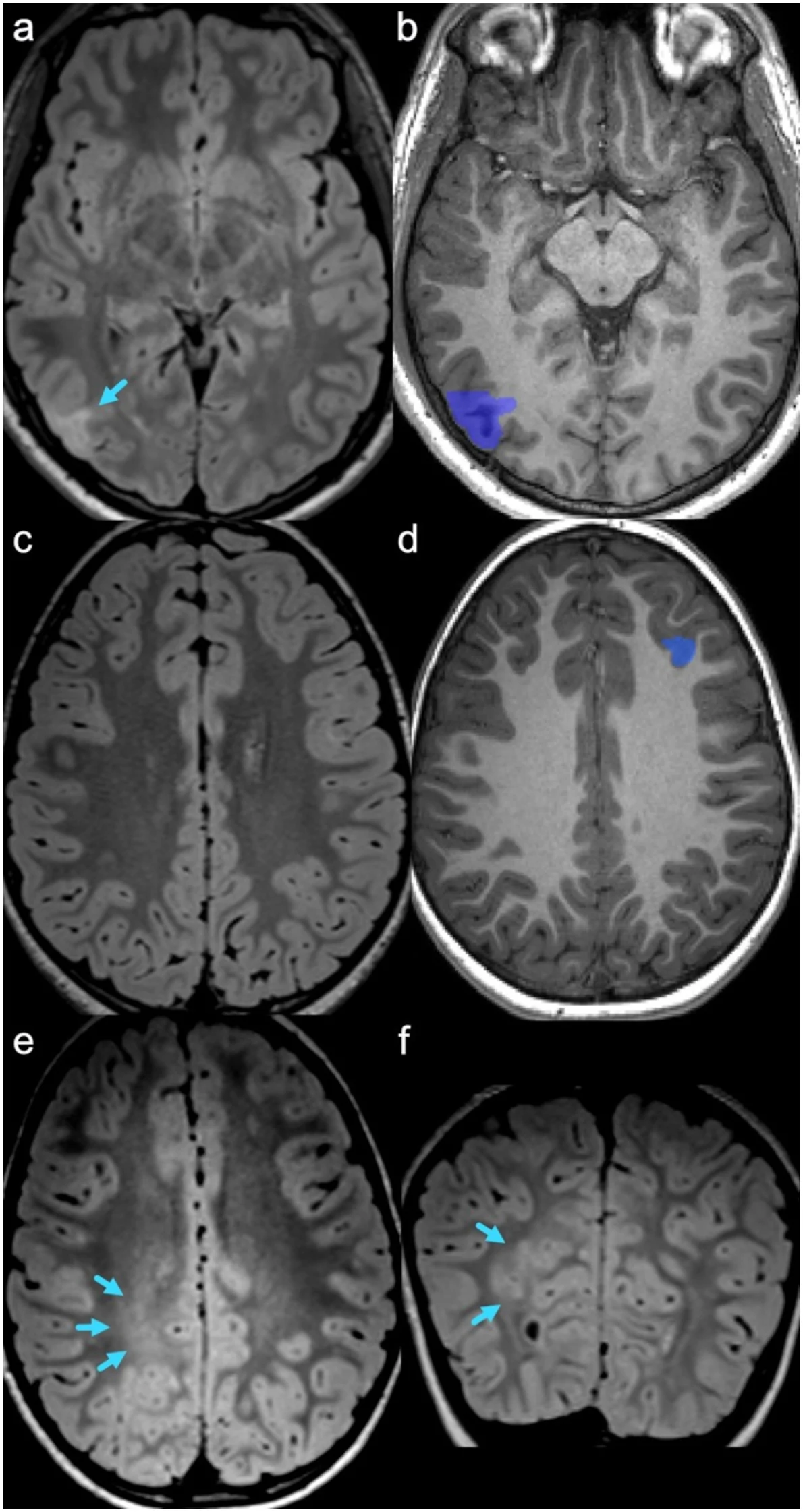
Representative imaging examples (3D-nnUNet): (a–b) Correctly detected lesion (TPL) in the right temporal lobe consistent with presumed FCD type 2B, demonstrated on FLAIR (blue arrow, a) with the corresponding 3D-nnUNet segmentation mask on the 3D T1-weighted image (b). (c–d) False-positive detection (FPL) by 3D-nnUNet (d) with the corresponding FLAIR image shown in (c). (e–f) Lesion not detected (FNL) by 3D-nnUNet but visible on FLAIR (blue arrows, e, f).

**Fig. 5:**
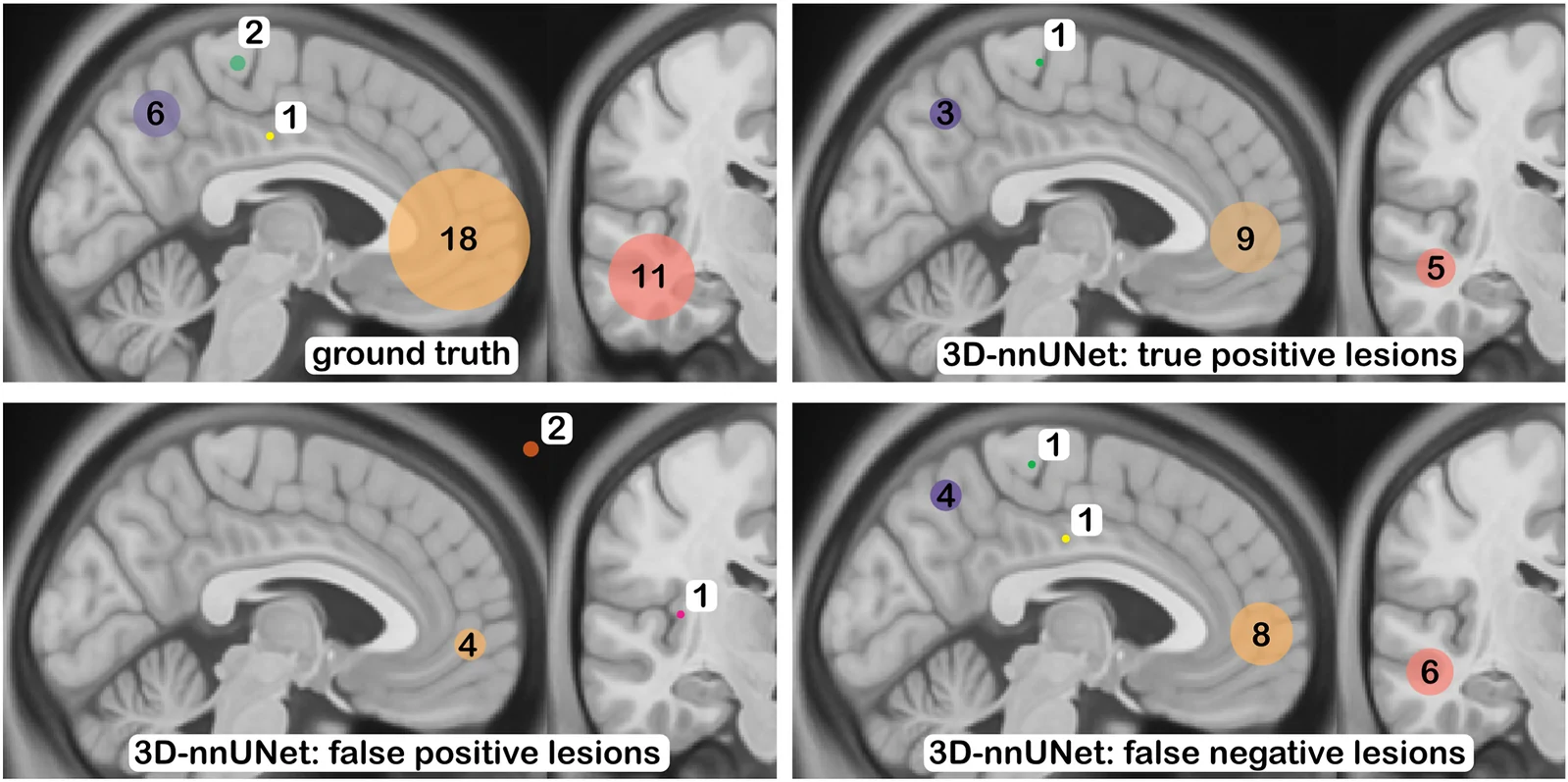
Comparison of lesion locations reported in the original radiology reports (ground truth) with corresponding 3D-nnUNet classifications as true positive, false positive, and false negative findings. Circle colors indicate anatomical region. Orange: frontal; light red: temporal; purple: parietal; green: frontoparietal; turquoise: occipital; yellow: cingulate, red: insula).

Examples for each category are given in Fig. 4, and the overall distribution is shown in Fig. 5.

### 3.5 Comparison between MELD Graph and 3D-nnUNet

At the lesion level, the two models showed substantial overlap, with 15 ground-truth lesions detected by both models and 11 missed by both. Among the discordant lesions, 9 were detected only by MELD Graph and 3 were detected only by 3D-nnUNet. At the patient level, both models correctly identified 15 of 34 MRIpos patients and correctly classified 16 of 36 MRIneg patients. MELD Graph additionally identified 9 MRIpos patients missed by 3D-nnUNet, whereas 3D-nnUNet additionally identified 3 MRIpos patients missed by MELD Graph. In the MRIneg group, MELD Graph produced false-positive classifications in 15 patients classified correctly by 3D-nnUNet, whereas 3D-nnUNet produced a false-positive classification in 1 patient classified correctly by MELD Graph.

### 3.6 Statistical evaluation

#### 3.6.1 Lesion detection

**Fig. 6:**
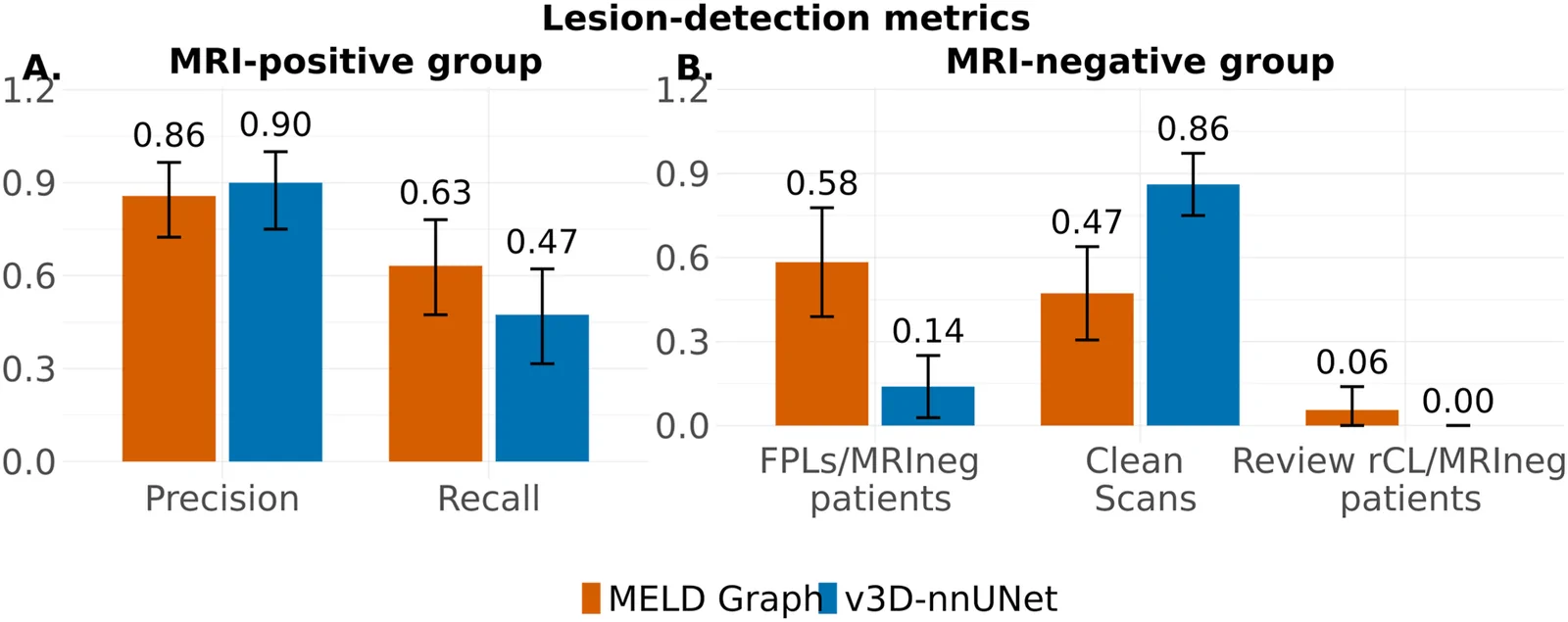
Metrics for the lesion detection task. A: In the MRI-positive group, both models showed comparable performance. B: In the MRI-negative (MRIneg) group, 3D-nnUNet produces fewer false positive lesion detections (FPLs) per MRIneg patient, likely reflecting a more conservative strategy derived from the neural network architecture and from the ensemble strategy. In contrast, MELD Graph identified candidate lesions(rCL) that were not described in the primary radiology report. The error bars represent 95% confidence intervals calculated using non-parametric bootstrapping with 1000 resamples.

In the lesion-detection task, the two models showed broadly similar performance within the MRIpos group. MELD Graph achieved a precision of 0.86 and a recall of 0.63, whereas 3D-nnUNet achieved a precision of 0.90 and a recall of 0.47. In the MRIneg group, MELD Graph produced 0.58 false-positive lesions per patient, whereas 3D-nnUNet produced 0.14 per patient. The proportion of MRIneg scans without false-positive detections was 0.47 for MELD Graph and 0.86 for 3D-nnUNet. Metrics for the lesion-detection task are shown in Fig. 6 and summarized in Table 3. Detailed counts are provided in Supporting Information Table S1.

#### 3.6.2 Patient classification

**Fig. 7:**
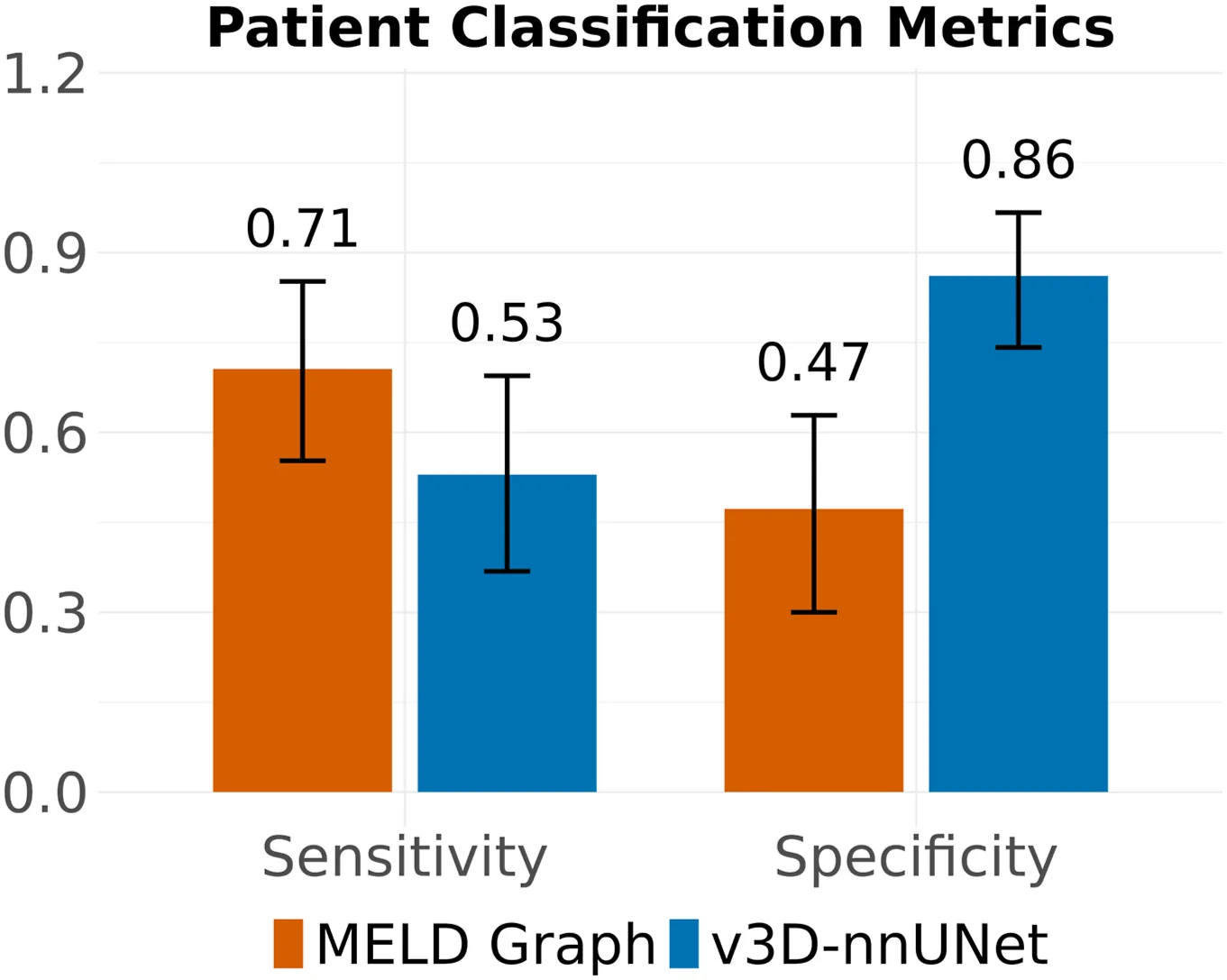
Patientwise metrics. The two models achieved similar sensitivity values, reflecting a comparable number of correctly classified MRI-positive patients, whereas 3D-nnUNet demonstrated higher specificity, reflecting a lower number of false-positive classifications (FPP). The error bars represent 95% confidence intervals calculated using non-parametric bootstrapping with 1000 resamples.

MELD Graph achieved a sensitivity of 0.71 and specificity of 0.47, whereas 3D-nnUNet achieved a sensitivity of 0.53 and a specificity of 0.86, reflecting the reduced number of FPPs. Metrics for patient classification are shown in Fig. 7 and summarized in Table 3.

#### 3.6.3 Statistical comparison

In the mixed-effects logistic regression model, no significant effect was observed for model (OR = 0.46, p = 0.114), FLAIR type (OR = 1.22, p = 0.72), age (OR = 1.05, p = 0.34) or sex (OR = 1.29, p = 0.62).

For the patient-level outcome, logistic regression revealed no significant associations with model, FLAIR sequence, age, or sex. Correct patient classification was higher for the 3D-nnUNet than for MELD Graph (OR = 1.66, p = 0.16), although this effect did not reach statistical significance. Age (OR = 1.05 per year, p = 0.15), FLAIR version (OR = 1.19, p = 0.76), and sex (OR = 1.01, p = 0.96) were also not significantly associated with patient-level performance.

**Fig. 8:**
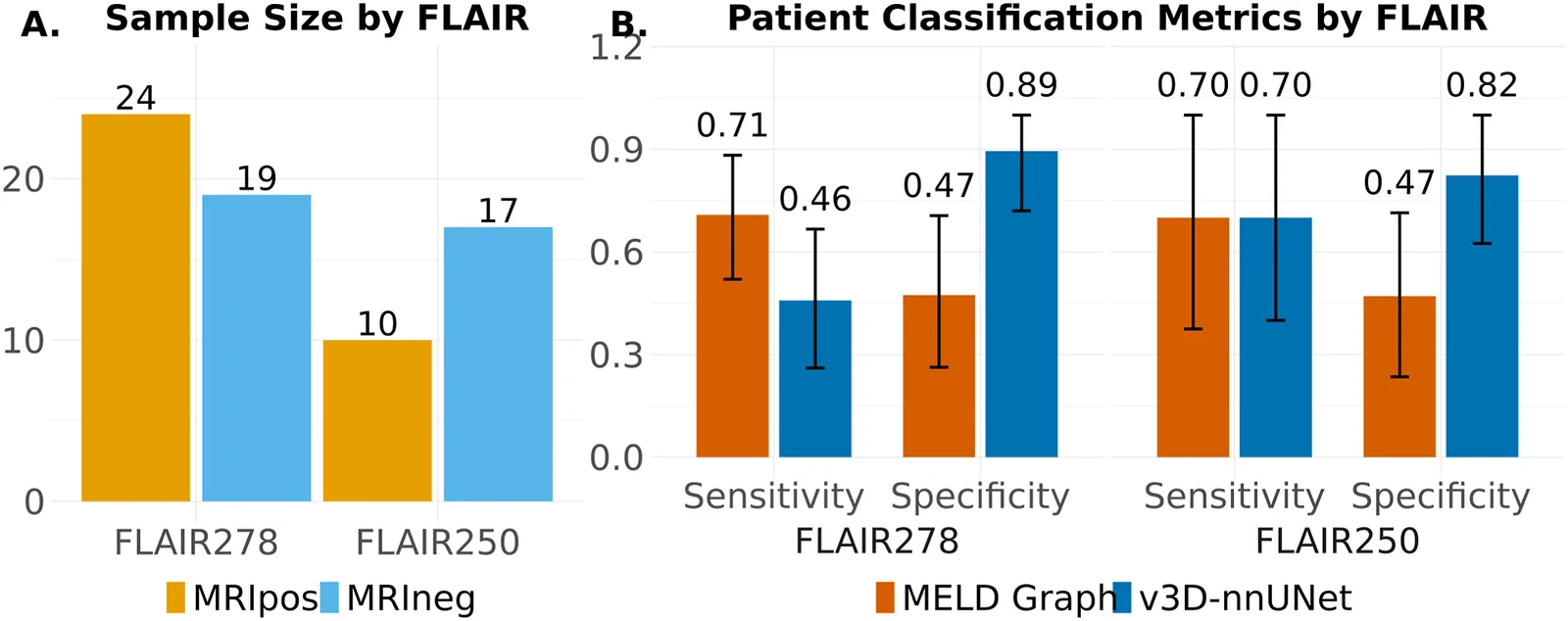
A. Sample size stratified by group and FLAIR version. The dataset contains fewer MRI-positive cases for the improved FLAIR250 sequence. B. The improved FLAIR sequence is associated with increased specificity for MELD Graph and increased sensitivity for 3D-nnUNet; however, these differences did not reach statistical significance. The error bars represent 95% confidence intervals calculated using non-parametric bootstrapping with 1000 resamples.

For false positives, 3D-nnUNet yielded significantly lower odds than did MELD Graph under the reference FLAIR condition (OR = 0.16, p = 0.006). Neither FLAIR250 (OR = 1.16, p = 0.755), nor the model-by-FLAIR interaction was significantly associated with a lower false-positive burden (OR = 1.89, p = 0.499). For false negatives, neither the model nor FLAIR showed a significant association (OR = 2.20, p = 0.10), and the interaction was not significant (OR = 0.45, p = 0.350). The results are summarized in Fig. 8.

## 4 Discussion

In this retrospective study, we externally validated two recent deep learning–based FCD lesion detection models, MELD Graph and 3D-nnUNet, in a cohort of children and adolescents. The central neuroradiological challenge is to identify a structural lesion that may explain the epileptogenic focus and guide appropriate therapeutic decision-making. As these lesions can be very subtle and because seizures and EEG abnormalities may also occur in structurally normal-appearing brains, we deliberately included MRIneg scans in our validation cohort.

In the lesion-detection task for the MRIpos group, the two models showed comparable performance. High precision indicates that most detected lesions represent TPL, with a small number of FPL findings in MRIpos patients. In contrast, moderate recall demonstrates that a substantial proportion of FCDs remains undetected, meaning that neither model can reliably exclude structural pathology. Expert neuroradiological review therefore remains indispensable. In their current form, the models should be regarded as decision-support tools rather than replacements for experienced neuroradiologists.

In the MRIneg cohort, MELD Graph generated more FPL detections than did 3D-nnUNet. This higher false-positive burden is clinically relevant, because each candidate lesion requires expert reassessment and may increase reporting time. A potential explanation for the observed differences in false-positive occurrence lies in the fundamentally different representations used by the two models. 3D-nnUNet operates voxelwise, relying on local image intensity patterns. This design favors segmentation only when the learned signal is sufficiently strong, resulting in more conservative behavior, particularly in MRIneg patients.

In contrast, MELD Graph operates on surface-derived features of the cortex. While this approach is well suited to capture cortical patterns typical of FCD, it may be more sensitive to developmental variability in the pediatric cortex. Moreover, MELD Graph relies on the harmonization of surface-based features across subjects. Although our sample size exceeded the approximately 20 subjects suggested in the original publication (Ripart et al., 2025), the rapidly evolving cortical morphology in pediatric populations may require larger age-stratified samples to achieve robust harmonization. In addition, FreeSurfer-based surface reconstructions are known to be less reliable in younger children and infants (Ghosh et al., 2010), which may further contribute to spurious surface abnormalities. MELD Graph applies the same FreeSurfer atlas across all ages. Nevertheless, our logistic regression analysis did not reveal an association between patient age and the occurrence of false positives for either method, suggesting that age alone does not fully explain the observed differences.

Notably, the ratio of false-positive lesions per patient was similar for 3D-nnUNet in the MRIpos and MRIneg groups. In contrast, MELD Graph produced a substantially higher number of false-positive lesions in the MRIneg group, and a similar trend can be inferred from the results reported by Kaas et al. (2026).

Our findings are consistent with previous work on automated FCD detection via both voxelwise and surface-based approaches, suggesting that both models can be generalized to a pediatric cohort. For MELD Graph, both precision and recall were broadly in line with the values reported in the original publication (Ripart et al., 2025). Compared with the original 3D-nnUNet study (Kersting et al., 2025), as well as a recent pediatric-only validation (De Vita et al., 2025) and a recent mixed-cohort validation (Kaas et al., 2026), our precision values were higher, whereas recall remained similar overall. An exception was Kaas et al. (2026), in which higher recall values were reported. In particular, the study by Kaas et al. included a subset of patients with structural abnormalities other than FCD, including tumors, mesial temporal sclerosis, cysts, gyral abnormalities, and infarction- or trauma-related sequelae, whereas such cases were excluded from our cohort, except for three lesions that were recognized only postoperatively as non-FCD pathologies because they could not be reliably distinguished from FCD on MRI. This difference in inclusion criteria may have influenced the pattern of false-positive results and therefore may have contributed to the discrepancies between studies.

Notably, the false-positive burden of both models was lower than that previously reported for FCD detection tools in adult and pediatric cohorts (Gill et al., 2021). For example, Gill et al. (2021) reported an average of six false-positive cases per patient. Moreover, Kaas et al. (2026) reported a substantially lower number of false-positive lesions for MELD Graph than for its predecessor, the MELD Classifier (Spitzer et al., 2022), or for DeepFCD (Gill et al., 2021).

In the patient-wise classification task, both MELD Graph and 3D-nnUNet achieved moderate sensitivity. This finding indicates that both approaches were able to correctly identify a substantial proportion of patients with FCDs while still failing to detect a nonnegligible fraction of affected patients, potentially delaying the necessary referral for epilepsy surgery. In contrast, 3D-nnUNet achieved higher specificity than did MELD Graph, consistent with the more conservative detection profile described above. Higher specificity may be advantageous in clinical practice, as it reduces unnecessary and time-consuming reviews, multidisciplinary discussions, or additional diagnostic investigations. The concordance data between the two models further illustrates that joining the two models into a combined decision rule would not necessarily improve overall performance. Based on the observed overlap, requiring both models to classify a patient as positive would increase specificity to 0.89 but reduce sensitivity to 0.44. Thus, joint model interpretation may be useful to identify high-confidence candidate lesions, but discordant detections should not be dismissed when the clinical aim is to maximize sensitivity for subtle FCD detection.

Two different FLAIR protocols with different turbo factors were used in our cohort. A lower turbo factor (FLAIR250) reduces T2-weighted blurring and improves signal stability, thereby enhancing grey–white matter differentiation and potentially improving lesion conspicuity and border delineation. Using MELD Graph version 2.2.2, higher-quality FLAIR250 images were associated with a marked increase in specificity, primarily due to fewer false-positive detections, whereas sensitivity remained largely unchanged. Interestingly, this difference disappeared after repeating the analysis with MELD Graph version 2.2.5, which incorporates minor FreeSurfer-recommended fixes to the surface reconstruction pipeline. This finding highlights the importance of accurate cortical surface reconstruction, as MELD Graph relies on surface-based features. In contrast, 3D-nnUNet demonstrated higher sensitivity with FLAIR250, whereas specificity changed only modestly. As a voxel-based model, 3D-nnUNet is expected to benefit directly from improved image quality. Higher signal-to-noise ratio reduces background variability and increases lesion-to-background contrast, making subtle FCD-related signal abnormalities more likely to be detected, even after resampling to the T1w space.

Although the primary radiology reports and the subsequent review by an expert pediatric neuroradiologist served as the reference standard, this approach has inherent limitations. FCDs can be highly subtle and difficult to distinguish from morphologically similar pathologies. In our cohort, three lesions initially presumed to represent FCD on presurgical MRI were later histologically confirmed as gangliogliomas or gliotic tissue. As neither MELD Graph nor 3D-nnUNet was trained exclusively on histologically confirmed FCDs, the models should not be regarded as histopathological classifiers. Instead, they identify imaging abnormalities suggestive of FCD that warrant closer review. Consequently, positive predictions may also occur in morphologically similar epileptogenic lesions, such as gangliogliomas or focal gliosis, which can be difficult to distinguish from FCD even for experienced neuroradiologists. Clinically, however, this is of limited importance, as the primary purpose of the models is to highlight subtle epileptogenic abnormalities rather than establish a definitive pathological diagnosis. Furthermore, many lesions classified as FCD were not confirmed histologically because surgery or biopsy was not performed. Consequently, the reference standard inevitably relied primarily on the original neuroradiological interpretation, and occasional discrepancies between model predictions and the reference standard may reflect uncertainty in the underlying diagnosis rather than true model errors. Specificity was assessed in MRI-negative epilepsy patients, a clinically relevant target population for automated FCD detection. However, the present study did not include healthy controls or a dedicated cohort with FCD-mimicking pathologies. This is an important limitation, as clinical specificity requires testing not only against normal or MRI-negative cases, but also against relevant differential diagnoses that may resemble FCD on MRI. Future studies should therefore include such cohorts to better establish the diagnostic specificity and robustness of automated FCD-detection tools in routine clinical practice.

Furthermore, the reevaluation of FPL detections remains subjective. In principle, both model architectures may identify abnormalities that are not readily appreciable via visual inspection. Two rCL were identified only after careful re-examination of all available sequences. Notably, neither of these findings correlated with the clinical findings, raising the possibility that model prompting may have influenced expert interpretation, a phenomenon known as automation bias, i.e., the tendency to over-rely on and uncritically accept automated recommendations. FPL and rCL may therefore actually not only decrease workflow efficiency but also increase automation bias if interpreted without sufficient clinical and electroencephalographic correlation but this phenomenon is not different from a setting involving multiple experts and complex diagnostic tasks, where a degree of subjectivity is unavoidable and must be acknowledged. This is clinically relevant because false-positive model outputs may not only increase review time, but may also create diagnostic uncertainty and, if overinterpreted, could contribute to unnecessary downstream investigations or ineffective treatment decisions. Another limitation affecting both the AI models and the human reader is the inherent uncertainty in defining the size and spatial extent of FCDs. Delineation of these lesions is challenging because lesion borders are often poorly defined, manual segmentations show considerable inter-rater variability, and the MRI-visible abnormality does not necessarily correspond to the epileptogenic zone or the eventual surgical resection margin (Wehner and Lüders, 2008). Consequently, lesion volumes and boundaries frequently differed between raters and algorithms, and minor discrepancies in lesion location have also been reported in the original MELD Graph study (Ripart et al., 2025). As the primary clinical role of MELD Graph is lesion detection and localization rather than precise lesion delineation, we considered lesion localization to be the clinically relevant endpoint and therefore did not evaluate voxel-wise overlap metrics such as the Dice similarity coefficient. Interestingly, Kersting et al. similarly complemented voxel-wise Dice with localization-based measures, including pinpointing and bounding-box Dice, emphasizing that clinically relevant performance extends beyond exact voxel-wise agreement (Kersting et al., 2025). Future studies comparing presurgical AI-based lesion localization, surgical resection margins, and postoperative seizure outcomes will be important to determine which evaluation metrics best predict clinical benefit.

Finally, this study is limited by its relatively small sample size. This can be especially important in MELD Graph where the feature harmonization step can be crucial for the occurrence of FPL. It remains uncertain whether including a larger number of subjects across different pediatric age groups would improve the robustness of the harmonization and, consequently, model performance.

In conclusion, our findings support the generalizability of both MELD Graph and 3D-nnUNet to an independent pediatric cohort. Both models demonstrated high precision in detecting FCDs within our pediatric cohort but only moderate sensitivity, leaving a clinically relevant proportion of lesions undetected. Both models therefore represent valuable decision-support tools that may enhance diagnostic confidence, yet they are not suitable as independent screening systems. Their output should always be interpreted in the context of clinical and electroclinical findings rather than accepted uncritically. The goal of AI in epilepsy imaging has to be to enhance expert perception by reducing overlooked subtle lesions while preserving independent clinical judgement.

## CRediT authorship contribution statement

**Andrea Dell’Orco:** Writing – review & editing, Writing – original draft, Visualization, Software, Methodology, Formal analysis, Data curation, Conceptualization. **Enrico De Vita:** Writing – review & editing, Software, Methodology. **Felice D'Arco:** Writing – review & editing, Methodology. **Annalena Lange:** Writing – review & editing. **Theodor Rüber:** Writing – review & editing. **Angela M. Kaindl:** Writing – review & editing, Resources, Investigation. **Mike P. Wattjes:** Writing – review & editing, Resources. **Ulrich-Wilhelm Thomale:** Writing – review & editing, Resources. **Lena-Luise Becker:** Writing – review & editing, Resources, Investigation. **Anna Tietze:** Writing – review & editing, Writing – original draft, Supervision, Methodology, Investigation, Data curation, Conceptualization.

## Statement on generative AI use

During the preparation of this work, the authors used generative AI to support language editing and to improve readability and grammar. After using this tool, the authors reviewed and edited the content as needed and take full responsibility for the content of the article. No scientific content, data analysis methods, or figures were generated by generative AI.

## Funding

AT and AD were supported by Stiftung Charité (Clinical Fellowship). AMK was supported by the Einstein Stiftung Fellowship through the Günter Endres Fond and the Federal Ministry of Education and Research (Bundesministerium für Bildung und Forschung, BMBF) as part of the German Center for Child and Adolescent Health (DZKJ). AL & TR are funded by the German Federal Ministry of Research, Technology and Space (epi-center.ai).

## Declaration of competing interest

None of the authors have any financial or nonfinancial interests to disclose.

## Data Availability

The raw MRI data analyzed during the current study are not publicly available due to German data protection regulations but are available from the corresponding author upon reasonable request. Defaced example FLAIR images illustrating the two acquisition variants used in this study, as well as the table derived from the neuroradiological evaluation, are publicly available in the OSF repository (https://osf.io/ag78q). In addition, a GitHub repository containing the analysis code, including an R-based Jupyter notebook with an example statistical evaluation, is available at https://github.com/0rC0/FCDSegmentValidation.
