# Supplementary material for: Deep learning-based detection of focal cortical dysplasia in children: External validation of the MELD Graph and 3D-nnUNet pipelines
Source: Neuroimage Rep. 2026 Sep 16;6(4):100410. doi: 10.1016/j.ynirp.2026.100410 (PMC13595142; doi:10.1016/j.ynirp.2026.100410)
Supplement: Supplementary file 1 [file mmc1.docx]

## Computational Burden:

MELD:

The MELD Graph pipeline was executed on the local (HPC) cluster. The cluster nodes were equipped with 64 AMD EPYC (Rome) CPU cores, 512 GB RAM, and 3 TB of storage. We ran the workflow through the SLURM workload manager using a batch script submitted with a single task (--tasks=1) and 32 CPU cores per task (--cpus-per-task=32). Memory was requested as 64 GB (--mem=64G) and each job was limited to 8 hours wall time (--time=8:00:00).

Computational burden was about 3.5 hours for the FreeSurfer part of the MELD pipeline and 45 minutes for the harmonization and prediction part

3D-nnUNet

3D-nnUNet was run on a local research machine equipped with an AMD Ryzen Threadripper 3970X (32-cores), an NVIDIA RTX 3090 GPU with 24 GB of VRAM and 128 GB RAM .

The computational burden was approximately 3 minutes per subject, including FLAIR-to-T1w coregistration (<1 minute), prediction using the five models (~1 minute), and ensembling (<1 minute).

## S.1. Formulas

$$Precision = \frac{TPL}{(TPL + FPL)}$$

$$Recall = \frac{TPL}{(TPL + FNL)}$$

$$Sensivity = \frac{TPP}{(TPP + FNP)}$$

$$Specificity = \frac{TNP}{(TNP+FPP)}$$

## Tables

Table S1: Summary of lesion-level and patient-level classification counts and performance metrics for MELD Graph and 3D-nnUNet

| **Task** | **Metric** | **MELD Graph** | **3D-nnUNet** |
| --- | --- | --- | --- |
| Lesion detection (MRI-positive patients) | True positive lesions (TPL) | 24 | 18 |
| Lesion detection (MRI-positive patients) | False positive lesions (FPL) | 4 | 2 |
| Lesion detection (MRI-positive patients) | False negative lesions (FNL) | 14 | 20 |
| Lesion detection (MRI-positive patients) | Retrospective candidate lesions (rCL) | 2 | 0 |
| Lesion detection (MRI-positive patients) | Precision | 0.86 | 0.90 |
| Lesion detection (MRI-positive patients) | Recall | 0.63 | 0.47 |
| Lesion detection (MRI-negative patients) | False positive lesions (total) | 21 | 5 |
| Patient classification (all patients) | True positive patients (TPP) | 24 | 18 |
| Patient classification (all patients) | False positive patients (FPP) | 19 | 5 |
| Patient classification (all patients) | False negative patients (FNP) | 10 | 16 |
| Patient classification (all patients) | True negative patients (TNP) | 17 | 31 |
| Patient classification (all patients) | Sensitivity | 0.71 | 0.53 |
| Patient classification (all patients) | Specificity | 0.47 | 0.86 |

## Figures

Figure S1: Heatmap of the FSQC output metrics. Patients are ordered by decreasing age. Visual quality control resulted in a borderline rating (QC = 2) for sub-FCD0053, sub-FCD0060, sub-FCD0072, sub-FCD0097, and sub-FCD0110.

Figure S2: Example of a borderline case. The cortex is slightly undersegmented, while the labels remain largely symmetrical.

Figure S3: Example from the visual quality control. The large temporal lobe FCD is not assigned to a segmentation ROI, which likely contributed to outlier FSQC values. However, the overall segmentation and cortical reconstruction are correct. The lesion was correctly detected by both MELD Graph and 3D-nnUNet.

Figure S4. Performance metrics recalculated for MELD Graph after excluding cases classified as borderline by the FreeSurfer quality control. Metrics improved slightly, indicating that some borderline cases contributed to false-positive or false-negative classifications. However, these cases were retained in the main analysis because the quality control did not support their exclusion and the overall performance changed only marginally.

Figure S5: Comparison of MELD Graph using T1w images only with 3D-nnUNet. In the MRI-positive group, the T1w-only MELD Graph analysis showed lower precision and recall compared with the main MELD Graph analysis using FLAIR as a second modality. In the MRI-negative group, the overall performance was identical to the T1w+FLAIR analysis; however, the distribution of false-positive lesions differed between the two analyses. Only 12 patients showed false-positive lesions in both MELD Graph evaluations, and most false-positive findings occurred in different locations. Patient-classification metrics a slightly higher number of false-positive classifications in MRI-negative patients for the T1w-only analysis, indicating fewer correctly classified MRI-positive patients.
